# Characterization of Damage Progress in the Defective Grouted Sleeve Connection Using Combined Acoustic Emission and Ultrasonics

**DOI:** 10.3390/s22218579

**Published:** 2022-11-07

**Authors:** Lu Zhang, Zhenmin Fang, Yongze Tang, Hongyu Li, Qizhou Liu

**Affiliations:** College of Civil Engineering and Architecture, Guilin University of Technology, Guilin 541004, China

**Keywords:** grouted sleeve connection, internal flaws, acoustic emission, ultrasonics, prefabricated building

## Abstract

The grouted sleeve connection is one of the most widely used connections for prefabricated buildings (PBs). Usually, its quality can have a significant impact on the safety of the whole PB, especially for the internal flaws that form during sleeve grouting. It is directly related to the mechanical performance and failure behavior of the grouted sleeve. Therefore, it is essential to understand the damage progression of the defective grouted sleeve connection. However, destructive testing is the mainstream measure to evaluate the grout sleeves, which is not applicable for in situ inspection. Therefore, this paper proposes a combined acoustic emission (AE) and ultrasonic testing (UT) method to characterize the damage progress of a grouted sleeve with different degrees of internal flaws under tensile loading. The UT was conducted before loading to evaluate the internal flaws. Additionally, the AE was used as the processing monitoring technique during the tensile testing. Two damage modes were identified: (i) brittle mode associated with the rebar pullout; (ii) ductile mode associated with the rapture of the rebar. The UT energy ratio was selected as the most sensitive feature to the internal flaws, both numerically and experimentally. The AE signatures of different damage phases and different damage modes were determined and characterized. For the brittle and ductile damage modes, two and three phases appeared in the AE activities, respectively. The proposed combined AE and UT method can provide a reliable and convenient nondestructive evaluation of grouted sleeves with internal flaws. Moreover, it can also characterize the damage progress of the grouted sleeve connections in real-time.

## 1. Introduction

In recent years, the applications of PB have boomed in China due to its cost-effectiveness, high efficiency, and labor-saving properties. Therefore, much attention from industry and academia has been drawn to studying overall performance [1,2,3]. Herein, the connection joints of PB play an important role in the mechanical characteristics, structural stability, and reliability of the entire building [4]. Moreover, the connection is directly related to the failure behavior. Many types of connection joints have been used in PB structures, e.g., bellows slurry anchor connections, thread sleeve connections, grouting sleeve connections. Among them, the grouted sleeve is one that is most widely used because of its reliability and efficiency in construction [5,6]. According to the connection mechanism, the grouted sleeve can provide sufficient adhesive force, so as to guarantee the integrity and stability of PB structures [7,8,9]. However, in practice, even by using nonshrinkage and high-strength grouting materials, many types of flaws and defects still exist due to leakage of grouting materials. Usually, those internal flaws and defects are impossible to avoid: undischarged air on the sleeve, operational errors in the construction process, mortar backflow, and so on [10]. Therefore, it is essential to develop an effective method to evaluate the internal flaws and monitor the damage progress in the grouted sleeve.

To this end, various methods and techniques have been adapted. Conventional destructive testing methods are the most commonly used, such as the pull-out method, core drilling method, and post-anchoring method. Though the destructive methods are very accurate and reliable, they can only directly give an evaluation of the internal flaws and damage process; those methods still have many issues. For example, it will damage the concrete structure and cannot be applied to the grouting sleeve connector in the PB structure, and most importantly, it cannot be adopted as on-site testing. The testing technology of grouting plumpness of sleeves is based on the embedded steel wire drawing method [11], vibration testing of an embedded damping transducer [12], and an endoscopy inspection through a drill hole in the grouted sleeve [13], similar to many destructive methods that involve low cost-effectiveness and complex post-operations. Thus, in order to meet the requirement of a rapid on-site test, some nondestructive tests were applied to the grouted sleeve. The radiography technique has been successfully used in the identification of internal flaws in grouted sleeves. Gao et al. detected the grouting compactness of sleeves based on industrial X-ray computed tomography (X-CT). The experimental results show that the method overcomes the interference problems from steel bars, concrete, and sleeves [14]. Likewise, Zhang et al. studied the quality of the sleeve grouting of a 200-mm-thick precast shear wall with portable X-rays. The experimental results showed that the proposed method was feasible for detecting the grouting compactness in both center and plum-pile arrangement sleeves [15]. He et al. used γ-rays to detect grouting defects in prestressed pipelines, and the results showed that it can clearly identify grouting defects [16]. Though digital radiography methods can provide accurate evaluations of internal flaws and soundness of concrete in a grouted sleeve, they require extensive safety attention and are expensive, which limits the potential application of this technology in on-site tests. Moreover, this method is usually only suitable for detecting parallel pieces and depends on the shielding conditions in the laboratory. 

There are many other nondestructive testing methods, for example, Tang et al. used a visual method to measure concrete-filled steel tube columns [17,18]. The results of the study showed that the vision measurement method can be used to accurately measure curved surface deformations, as well as to evaluate the full field strain in the recycled aggregate concrete-filled steel tube columns. Ahmadi et al. tried to improve the reliability of detecting and identifying structural faults by proposing new signal methods for damage detection of pedestrian truss bridges and concrete piers [19,20,21]. The results showed that the proposed method and damage index could identify damage and achieve highly accurate localization. Liu et al. used the impact echo method to detect the grouting density of sleeves, but its accuracy was low and it was only used for qualitative evaluation [22]. Luo et al. used the infrared thermal imaging method to detect the grouting fullness of the sleeve [23]. Wu et al. used the ground-penetrating radar (GPR) method to detect the grouting defects [24]. Similarly, the applicability of them is limited due to low accuracy. Due to this, the UT method has many merits (e.g., high efficiency, low cost, and sensitivity to microstructure), and is used for inspecting the flaws in the grouted sleeve. Li et al. used the UT method to detect the grouting defects in sleeves. The experimental results showed that the ultrasonic testing method can effectively detect grouting defects [25]. Yan et al. used the UT method to detect the grouting defects in sleeves. Through the analysis of the sound velocity value of the ultrasonic detection of the grouting sleeve, the ultrasonic detection can effectively detect the position of the grouting defect in sleeves [26]. Liu et al. used the UT method to detect grouting defects in sleeve joints in PB structures. The results showed that the proposed ultrasonic testing method can be used to test the grouting quality of sleeve joints in field PB structures [27]. According to the research of the appellate scholars, the ultrasonic detection method for detecting the grouting defects of sleeves has the advantages of high efficiency, accuracy, and convenience for on-site use. However, the ultrasonic testing method is not applicable for monitoring progress. As mentioned above, though, the infrared thermal imager method, optical fiber sensor method, and the potential monitoring method are capable of realizing, processing, and monitoring their use is limited because of their low accuracy. AE methods can also be used to predict and characterize various damage mechanisms of structures in real-time. Huijer et al. used acoustic emission methods to monitor carbon fiber-reinforced composites [28]. Šofer et al. conducted damage analysis of composite CFRP tubes using acoustic emission, and a pattern recognition approach was used to determine the damage modes [29]. Jeong et al. used AE to predict fractures during the sheet metal forming process [30]. Nazaripoor et al. detected the damage progress in short glass fiber-reinforced composite panels by using AE [31]. Wang et al. used an acoustic emission localization concept to understand the energy distribution in the embedded section of a steel bar during the pull-out test of a steel bar and a piece of concrete [32]. While, it shows more advantages in its high efficiency, low cost, and high applicability [33,34,35,36,37], the AE has a wide range of applications in the field of civil engineering. Boniface et al. used acoustic emission methods to monitor mechanical damage in concrete [38]. Wu et al. used the AE monitoring method to characterize the damage process of brick masonry during uniaxial compression [39]. Shahidan et al. used the AE monitoring method to classify the damage grades of reinforced concrete beams [40]. 

For the grouted sleeve, Joel E. Parks et al. used the AE monitoring method to characterize the damage process of the grouted splice sleeve during monotonic tension [41]. Han et al. used the AE monitoring method to qualitatively evaluate the bond failure process of the grouting sleeve, and the rapid increase in the ringing count may be a precursor to the bond failure of the grouting sleeve [42]. All the reported cases can prove that the AE monitoring method has better applicability in monitoring the damage process. Though AE has been successfully used in many different types of structures for identifying the damage process, the mechanism of the AE sources is still challenging. Other than that, multiple sensing techniques can overcome the drawbacks of each NDE method and greatly increase the reliability of measurements. 

By exploring engineering applications, the grouted sleeves may exhibit various parameters related to the defects, including location, length, thickness, and number. Other than that, the grout direction, rebar eccentricity, and grout type may also exist [43]. Herein, one of the most common defects was the horizontal defect; moreover, Guo et al. [44] found that the horizontal defect can significantly impair the mechanical performance of the grouted sleeve connector in PB. Therefore, this paper mainly focuses on the effect of horizontal defects in the grout sleeve.

Therefore, this paper proposes to combine AE and UT to provide the quantitative evaluation of the internal flaw of the grouting sleeve, and realize the real-time monitoring of the damage process of the grouting sleeve with different levels of internal flaws. Furthermore, different failure modes of the grouting sleeves can be characterized by extracting and analyzing the AE characteristic parameters associated with the mechanical properties and offline UT results. The outline of this paper is as follows: The feasibility of combined UT and AE detection is presented in Section 2. The mechanism of detection is verified numerically and experimentally in Section 3, Section 4 and Section 5, respectively. The damage processing monitoring performed by AE is presented in Section 6. The conclusions are summarized in Section 7.

## 2. The Mechanism of Ultrasonic-Based Detection on the Internal Flaws

**Figure 1:**
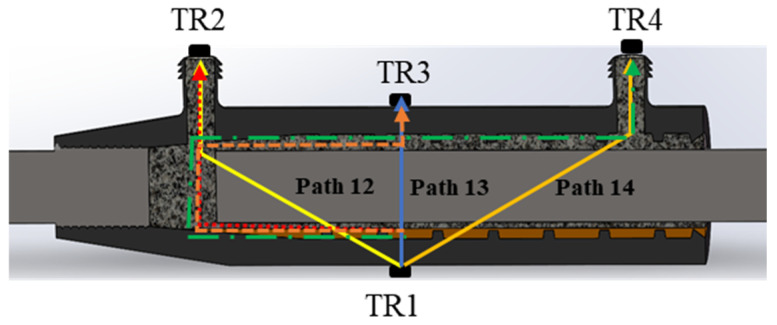
Grout defect detection system based on UT wave measurement.

In order to illustrate the mechanism of the ultrasonic-based method in the detection of internal flaws in the grouted sleeve, theoretical and numerical analysis were conducted in this section. The UT method is based on the propagation of an ultrasonic wave. The targeted objects (e.g., flaws, cracks, material change, deterioration, etc.) can disturb the ultrasonic wave propagation, which will reflect on the change in the UT signal. Though the mechanism is straightforward, the signal change could be caused by multiple factors, and the path of the wave propagation is varied. Therefore, we need to understand that the main reason causing UT signal changes is the internal flaw due to the lack of grouting, which is essential to determining the proper UT wave propagation path and feature. Firstly, three assumptions were made: (i) the path of ultrasonic wave propagation is straight; (ii) the only first arrival wave pocket was considered; and (iii) scattering and absorption were ignored. Therefore, to make sure that the UT wave can pass through the grout defect area, with consideration of the layout of the grouted sleeve, the arrangement of the transducers is shown in Figure 1. In this case, the transmission mode was adapted: each transducer was used as a transmitter and receiver. Four UT transducers with an application frequency of 200 kHz were attached. In order to illustrate the wave propagation, the excitation signal with a certain frequency signal was applied to the UT transducer 1 (TR1). The other three UT transducers and receivers were arranged on the surface and the grouting inlet and outlet of the sleeve forms multiple propagation paths for the UT waves through the defect area. In order to better detect the grouting defects of the sleeve, the propagation paths are mainly discussed as Path 12, Path 13, and Path 14, including reflection and refraction that occurred on the interface of different materials. 

Taking the grouted sleeve with the internal flaws as an example, there are four-phase medium, sleeve, internal flaws (void, lack of grout), steel bar, and mortar in the sleeve. Each of the two mediums can form a boundary, and when the excited UT waveform passes through the boundary, reflection and refraction will happen. The partial energy will be transmitted into the other medium; but most of the energy will be reflected due to the different acoustic impedance. The mechanism can be illustrated by calculating the reflection coefficient (R) and the transmission coefficient (T) between all the possible boundaries, see Table 1. Moreover, the calculation is shown in Equations (1)–(3). 

**Figure 2:**
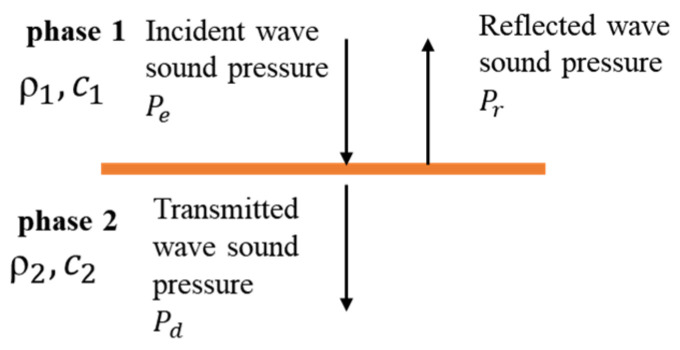
Calculation example: sleeve-plasticine interface.

According to Formula (1), the acoustic impedance of the UT wave propagating in different mediums in the sleeve is calculated, the reflection coefficient is calculated with Formula (2), and the transmission coefficient is calculated with Formula (3). As shown in Figure 2, it is possible to calculate how much energy is reflected back and how much energy is penetrated at each boundary within the sleeve.

(1)  Z=ρc 

(2) $$R=\frac{P_{r}}{P_{e}}=\frac{Z_{2}-Z_{1}}{Z_{1}+Z_{2}}\;$$

(3) $$T=\frac{P_{d}}{P_{e}}=\frac{2Z_{1}}{Z_{1}+Z_{2}}$$

where $Z_{i}$ is the acoustic impedance of a material, in this case, the wave is transmitted from phase 1 to phase 2, which is represented as subscripts 1 and 2, respectively. ρ is the density, c is the velocity of longitudinal waves. R is the reflection coefficient, $P_{r}$ is the reflected wave sound pressure, and $P_{e}$ is the incident wave sound pressure. T is the transmission coefficient, $P_{d}$ is the transmitted wave sound pressure. According to the calculation results in Table 1, most of the UT energy is reflected due to the larger reflection coefficient of the UT wave energy at each interface, and the change in the UT energy is the most obvious. To this end, the UT wave energy ratio can be used to quantitatively evaluate the defects. Most of the energy is reflected, the transmitted UT energy can be negligible. Therefore, the significant path of UT wave propagation is marked in Figure 1. For Path 13, the propagation mode is linear due to the vertical incident wave. However, the transmitted energy is relatively low. Both Path 12 and Path 14 can pass through the area with a flaw. However, Path 13 is longer than Path 12, which may introduce more complexity. Therefore, Path 12 is considered the most sensitive and best path. 

## 3. Numerical Validation

**Figure 3:**
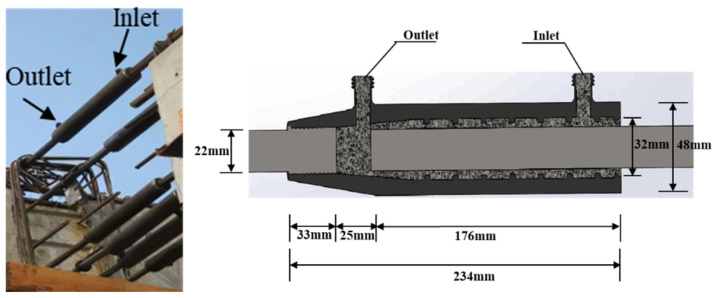
The details of the grouting sleeve.

**Figure 4:**
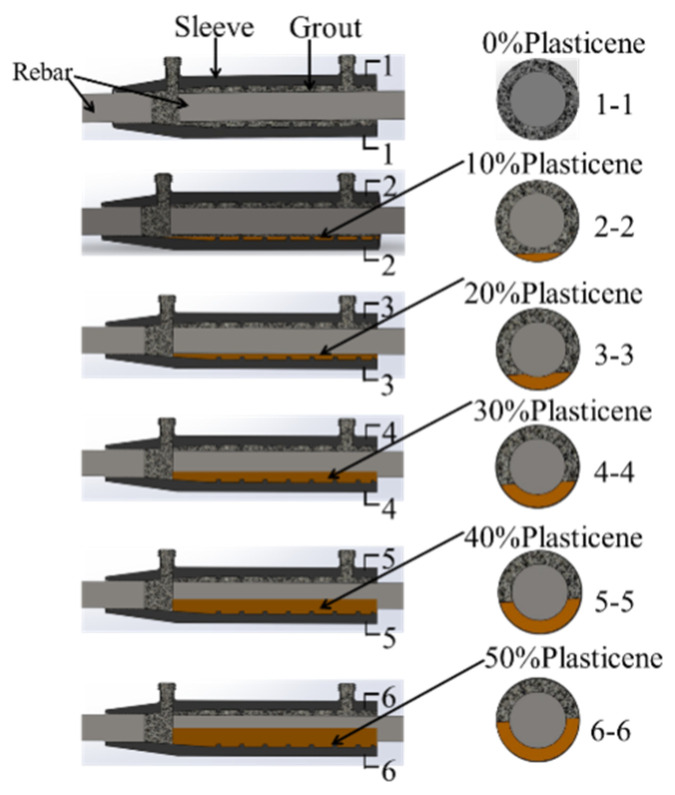
The grouting sleeve with different flaws.

**Figure 5:**
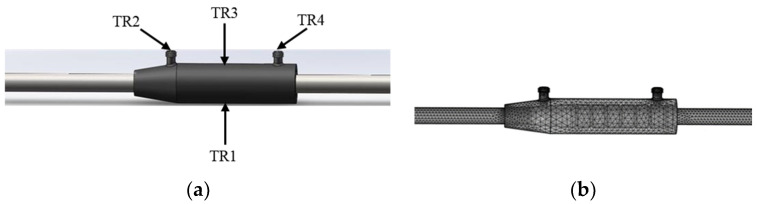
Numerical simulation: (**a**) FEA model and transducer setup; (**b**) meshing.

The grouting casing often has horizontal defects due to irregular operations. Especially for the beam-column or beam-beam connection, the in situ grouting implementation is shown in Figure 3. The most commonly used grouting sleeve is modeled, and its detailed dimensions are shown in Figure 3. In order to simulate the internal flaws, artificial flaws were introduced by adding oily plastic clay material. Six models of grouting sleeves with different volumes of plastic clay were established, as shown in Figure 4, and one transducer was attached to the surface of the sleeve as a transmitter, and the other three transducers were receivers attached to the grouting inlet, outlet, and opposite surface of the transmitter, as shown in Figure 5. In order to simplify the model and reduce the computational cost, the transducer was not included in the model; instead, the averaged displacements over the transducer-contacted area were used to represent the input and output response.

**Figure 6:**
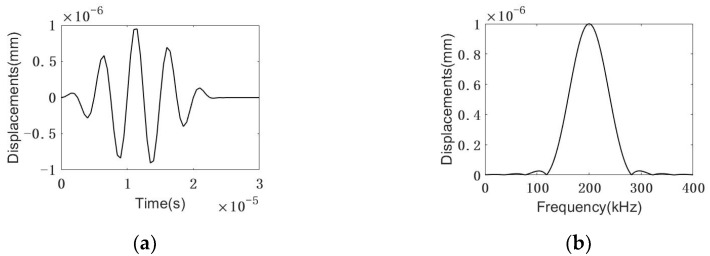
The excitation signal (**a**) in time domain, (**b**) in the frequency domain.

The numerical model was built using COMSOL Multiphysics 5.5 software. Table 2 lists the material properties. The mesh size of 0.162 mm and a time step of 0.25 μs were adopted, respectively, to achieve the 400 kHz resolution of wave propagation. Five-cycle tone burst signals modulated by the Hanning window with a frequency of 200 kHz were used as excitation. The transducer was not considered in the model for simplification. Therefore, the amplitude of the displacement input over the transducer-contacted area of the excitation signal was selected to be 1 nm (the same magnitude as 1 volt of electrical excitation), see Figure 6.

## 4. Numerical Results

**Figure 7:**
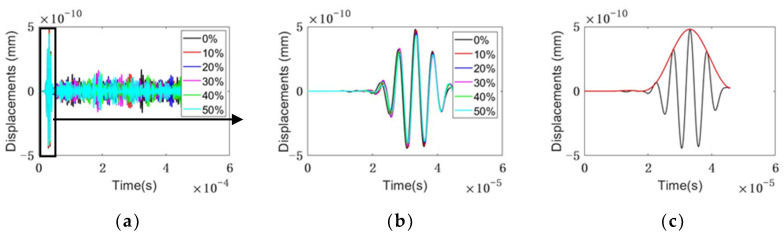
The time domain of Path 13: (**a**) The time domain; (**b**) the first wave; (**c**) an example of a Path 13 signal in the time domain indicating the envelope (red line) to calculate the ultrasonic energy.

**Figure 8:**
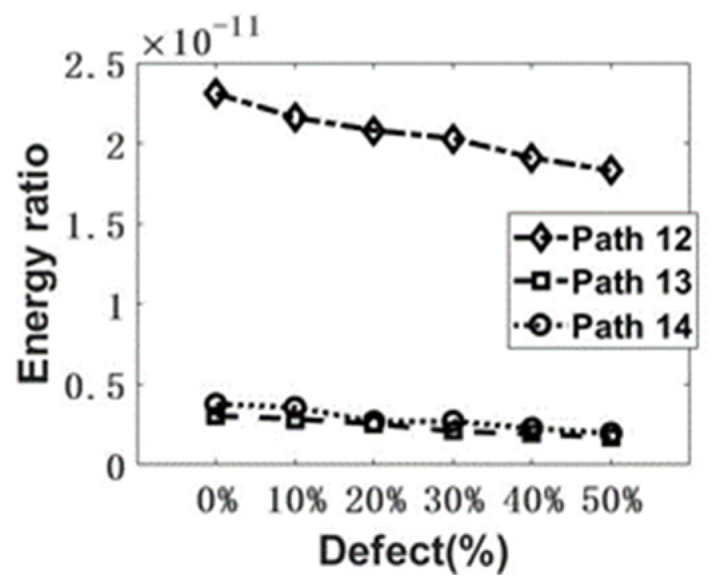
The numerical comparison of the UT signal energy ratio among the Path 12, Path 13, and Path 14.

The energy of the signal envelope [46] was obtained using MATLAB signal processing. Taking the signal from Path 13, for example, the time-domain signals from different samples are shown in Figure 7a,b. The calculation procedure is illustrated in Figure 7c. The energy of the signal envelope of the first arrival wave is calculated and summarized in Figure 8. As shown in Figure 8, for the response to 200 kHz, the transmitted UT energy decreases with the increase in the defect rate. Compared with Path 13 and Path 14, Path 12 is the most sensitive propagation path. The change in the UT energy ratio is the most significant among the three paths.

## 5. Experimental Verification

### 5.1. The Preparation of Specimens

**Figure 9:**
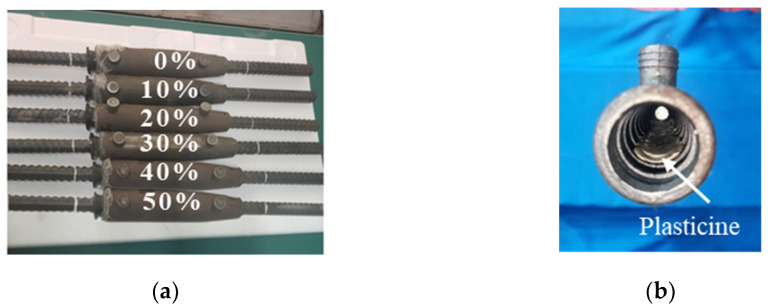
(**a**) grouting sleeve defect specimen; (**b**) schematic diagram of internal defects of sleeve.

In order to verify the theoretical analysis and numerical model, six samples with the same internal flaws as the numerical models were fabricated, see Figure 9a. Artificial defects were designed to simulate grouting defects in horizontal grout sleeve connectors due to leakage or under-grouting. The defects were long and strip-shaped and were manufactured between the bottom of the sleeve and the reinforcement. Since plasticine has the advantages of good plasticity, excellent bonding properties, and does not react with grouting materials, by adding plasticine to the grout sleeve, it can perfectly simulate artificial defects, as shown in Figure 9b. The different volume of plasticine was added to mimic the flaw rate. The volume ratio of plasticine to the capacity of the mortar in the grouted sleeve is marked in Figure 9 and used to represent the degree of the internal flaw. The position of the internal flaw is the same as the numerical model, see Figure 4. The grouting sleeve specimens with different defect rates are shown in Figure 9.

### 5.2. Experimental Setup

**Figure 10:**
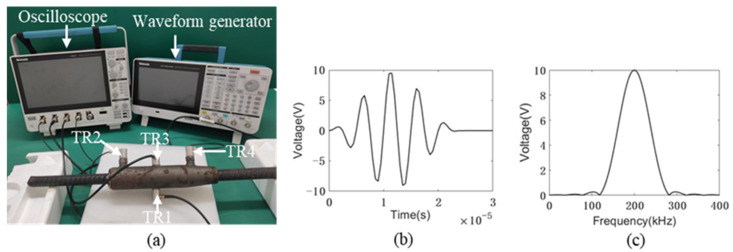
The setup of the active grout defect detection system: (**a**) experimental setup; (**b**) the excitation signal in time domain; and (**c**) the excitation signal in the frequency domain.

The same transducer arrangement as the numerical and theoretical models was adopted. The experimental setup is shown in Figure 10a. In this case, the same configuration as the numerical model was repeated. Transmission mode was used. Four transducers with a central frequency of 200 kHz were attached to the grouted sleeves as transmitters and receivers, see Figure 10. A tone burst signal with 5 cycles is generated by the waveform generator as an excitation signal with an amplitude of 10 V, see Figure 10b,c. Additionally, all the signals were collected by the oscilloscope. A 10 MHz sampling frequency was used.

### 5.3. Experimental Result

**Figure 11:**
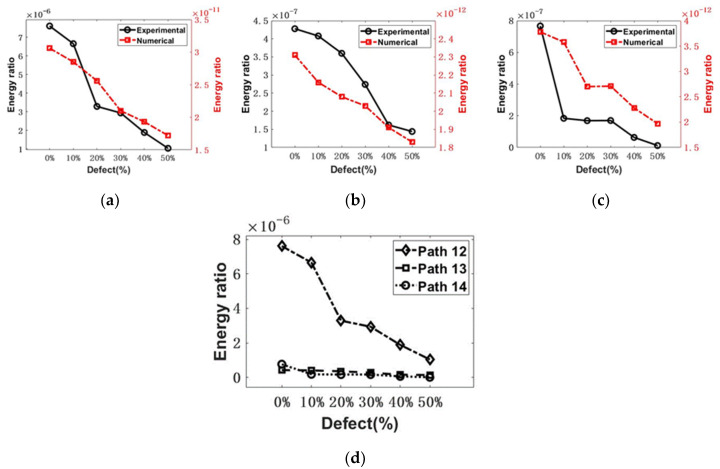
The comparison of UT signal energy ratio between experimental and numerical study from (**a**) Path 12, (**b**) Path 13, and (**c**) Path 14, (**d**) the experimental comparison of UT signal energy among Path 12, Path 13, and Path 14.

The time-domain signal collected in the experiment was filtered with the Butterworth bandpass filter, ranging from 10 kHz to 400 kHz, using the MATLAB software to reduce the EMI noise and other unexpected noise. The detailed signal processing method was described in Section 4, and the signal energy of the first arrival envelope was calculated. The comparison of numerical and experimental results is shown in Figure 11. The experimental results agree well with the numerical results, but still, differences can be observed because the transducer was not considered in the numerical model and some uncertainties rose. Similar to the numerical results in Section 4, the received UT energy decreases with the increase in the defect rate. It confirmed the mechanism of UT detection. The most sensitive path is Path 12 in this scenario. Moreover, before conducting UT, the proper experimental configuration should be determined: (i) the transducer should be placed to form the UT propagation path across the area with possible defects or flaws; (ii) the relatively simple path is highly recommended because a more complex path will bring uncertainties to the signal interpretation. Therefore, for evaluating the grouted sleeve, one transducer close to the grouting inlet and the other one in the middle of the sleeve is recommended to be optimum if the position of the internal flaw is unknown.

## 6. AE Characterization of Damage Progress in Grouted Sleeve Connector

### 6.1. Experimental Setup

**Figure 12:**
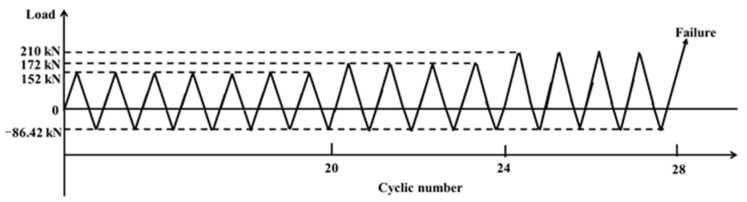
Loading procedure.

**Figure 13:**
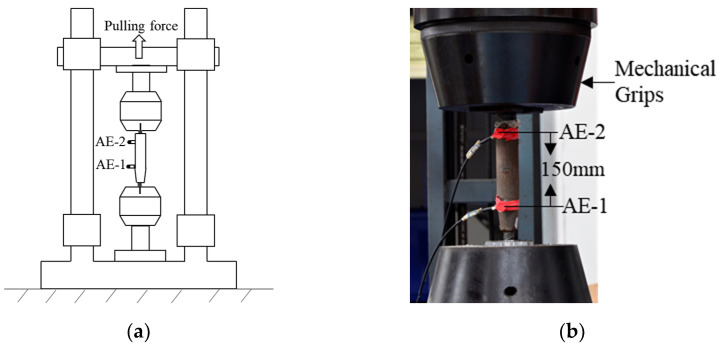
Test setup: (**a**) test setup design; (**b**) experimental setup.

The AE monitoring test pieces are six grouting sleeves with different defect rates, as shown in Figure 9a, and the material properties of the test pieces are shown in Table 2. The specific cyclic loading mode presented is adopted as required in ISO 15,835 [47], as shown in Figure 12. As shown in Figure 13a, the clamping length of the grouting sleeve rebar is 150 mm. The grouting sleeve is loaded by the high-speed impact testing machine.

The AE instrument adopted the Sensor Highway III of the American Physical Acoustics Company. The system is used to monitor the cyclic loading test of the grouting sleeve. As shown in Figure 13, two piezoelectric ceramic sensors, PK151 are used for source positioning. Sensor 2 is placed at the grouting inlet, named AE-2, and sensor 1 is placed at the slurry outlet, named AE-1. The sensor is coupled with hot melt adhesive and connected to a 26 dB gain preamplifier. The main data acquisition variables are the analog filters between 5 kHz and 400 kHz, the peak definition time is 300 µs, the hit definition time is 600 µs, the hit locking time is 1000 µs, and the threshold is 45 dB, which depends on the hit rate. The AE waveform is recorded at a sampling rate of 1 MHz, and the maximum duration is 1000 ms.

### 6.2. Grouted Sleeve Connector Cyclic Loading Test Results and AE Monitoring

**Figure 14:**
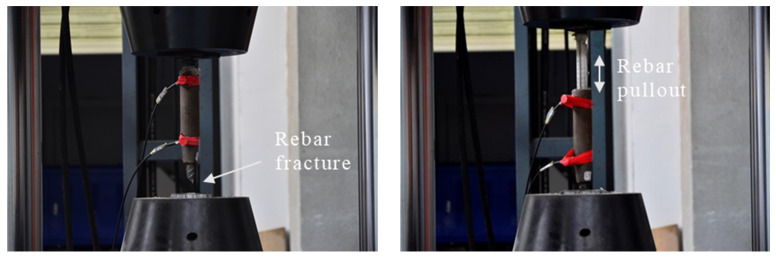
The two typical failure mode of the grouting sleeve connector: (**a**) rebar fracture; (**b**) rebar pullout.

**Figure 15:**
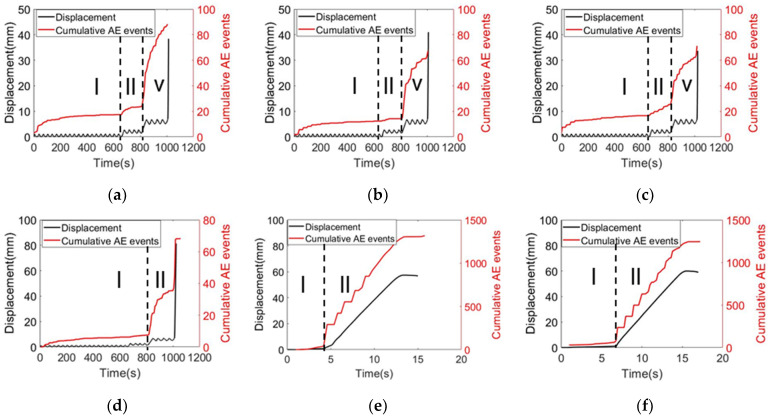
AE cumulative event history: (**a**) 0% defect; (**b**) 10% defect; (**c**) 20% defect; (**d**) 30% defect; (**e**) 40% defect; and (**f**) 50% defect.

The same cyclic loading for six samples is conducted and shown in Figure 14. Additionally, all the specimens were tested for failure. In general, two failure modes were observed: (i) rebar fracture; and (ii) rebar pullout. The detailed information is summarized in Table 3. The entire process of the testing was monitored by AE. The AE signals collected from the cyclic testing process were synchronized with the displacement response. In this case, the AE events were used to represent the AE activities. Figure 15 shows the relationship between the cumulative AE events and the displacement response. The AE activities show similar trends as the displacement response, which can reflect mechanical behavior. Firstly, the prescribed internal flaws played a crucial role in determining the failure mode: the ultimate failure mode of the rebar fracture corresponds to defect rates of 0%, 10%, and 20%; and the rebar pullout happened in the sleeve with defect rates of 30%, 40%, and 50%. 

Moreover, the damage progress also shows different patterns: ductile damage for rebar fracture and brittle damage for the rebar pullout, respectively. It reflects on the corresponding AE event history. As mentioned in the previous section, the internal defects or flaws are the decisive factor. For rebar breakage, three stages can be observed: as shown in Figure 15a–c. In the first stage, AE cumulative events are caused by friction between mechanical fixtures and steel bars, elastic compaction of high-strength concrete grout materials, and other unexpected uncertainties. In the second stage, due to the yield of reinforcement and the full development of the grouting crack, the AE cumulative event curves the mutation and rises slowly. In the third stage, a large number of acoustic emission signals were observed due to the necking and fracture of steel bars. AE cumulative events increase sharply.

For pullout damage, it shows brittle behavior as shown in Figure 15d–f. Unlike the rebar fracture, the damage progress can only be divided into two stages: the first stage is similar to the rebar fracture scenario, only few AE activities happened and were caused by friction or testing uncertainties. In the second stage, the jump of AE signals was observed. Most of signals were caused by concrete crushing. Since the internal flaws exist, the bonding strength among mortar, sleeve and rebar in the grouted sleeve reduced, leading to concrete crushing. As the interlock between steel bar and grout material losses, the rebar is pulled out quickly. For Figure 15e,f, the prescribed internal flaws already changed the entire mechanical performance of the grouted sleeve. Its tensile capacity is relatively low compared to the 30% defect case due to massive volume of the flaws. The damage grows very fast to failure. During this process, massive AE activities were observed. AE signals were caused by various sources (e.g., friction between rebar and mortar, concrete cracking and crushing). Under the same loading rate, for the sleeve defect rate of 0%, 10% and 20% of the specimens, the sleeve, mortar and rebar have good bonding connection performance, and rebar damage is the main damage. Rebar can be deformed under a certain bearing capacity, ultimately leading to the damage. This process was dominated by the ductile behavior. On the other hand, for the sleeve defect rate of 30%, 40% and 50% of the specimens, the sleeve, mortar and steel bonding connection performance is poor, mortar damage is the main form of damage. Mortar damage is the brittle damage, only few rebar deformations can be observed before eventual damage (concrete crushing). Therefore, the damage behavior corresponds to the slope of the cumulative AE event history. In the brittle damage process, the AE cumulative number increased dramatically during the second stage due to concrete crushing caused by rebar pullout and the third stage disappeared, no rebar fracture damage was observed, as shown in Figure 15. As a result, a rapid increase in the AE cumulative number of events may be a precursor of sleeve failure.

### 6.3. AE Localization of Damage

**Figure 16:**
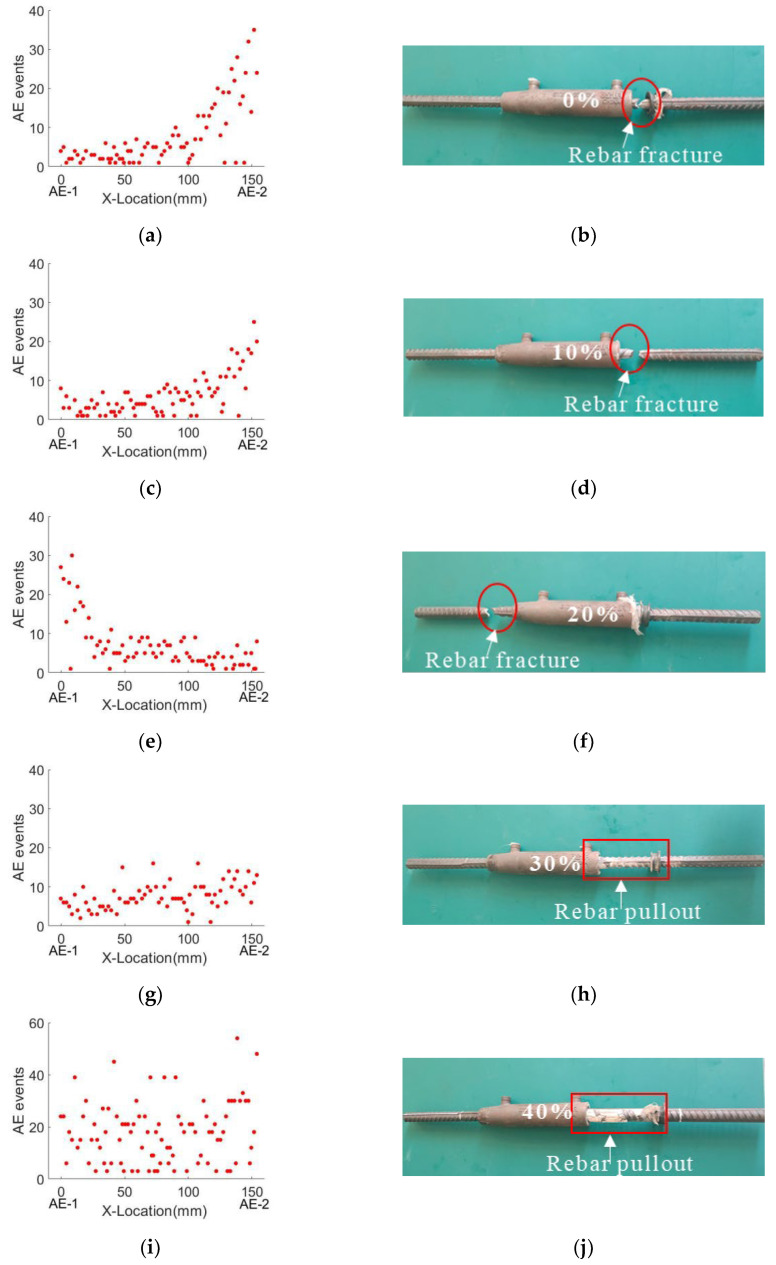

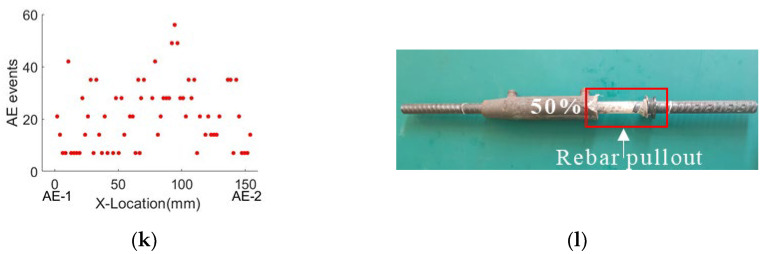
AE event distribution versus specimen failure diagram: (**a**,**b**) 0% defect; (**c**,**d**) 10% defect; (**e**,**f**) 20% defect; (**g**,**h**) 30% defect; (**i**,**j**) 40% defect; and (**k**,**l**) 50% defect.

As described in Section 6.2, under cyclic loading experiments, the six specimens with different defect ratios have two failure modes: the failure modes of rebar fracture correspond to 0%, 10%, and 20% defect rates; the rebar pullout happens in the sleeve with defect rates of 30%, 40%, and 50%. To further locate the location of the damage, a 1D linear localization method was used with the AE-1 (grouting outlet) sensor as the x-axis origin and the AE-2 (grouting inlet) sensor as the x-axis end point. The acoustic emission localization results, as shown in Figure 16, use the AE event number distribution to locate the damage location. For rebar-breaking failure, a large number of acoustic emission signals were recorded due to the necking and fracture of steel bars. At the location of failure, the number of AE events increases sharply, as shown in Figure 16a,c,e. For specimens with a 0% and 10% defect rate, the steel bar fracture occurred near the grouting inlet, as shown in Figure 16b,d. For the specimen with a 20% defect rate, the steel bar fracture occurred near the grouting outlet, as shown in Figure 16c. The failure position is consistent with the acoustic emission localization results. For rebar pullout failure, the acoustic emission signal mainly comes from the friction between mortar and steel bars and the breakage of mortars. The number of AE events mainly occurred between the AE-1 and AE-2 sensors, as shown in Figure 16g,i,k. The failure signal and friction signal of the mortar mainly occurred between the grouting inlet and the grouting outlet, as shown in Figure 16h,j,l. The damage location is consistent with the acoustic emission localization result.

### 6.4. AE Signatures of Damage Progress

**Figure 17:**
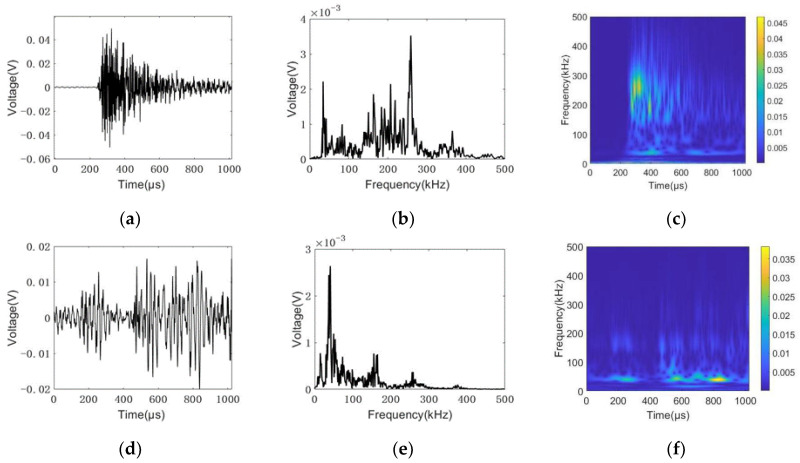

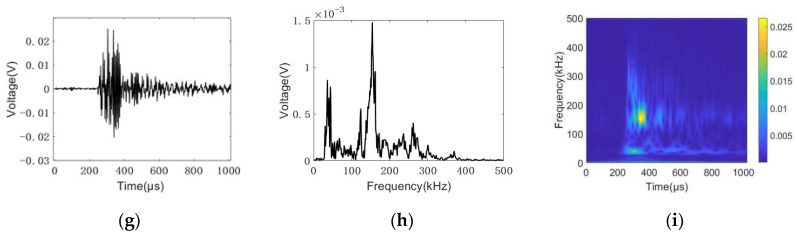
Time-domain histories, frequency domain histories, and frequency spectra of the AE signals detected by PK151 sensors, where (**a**–**c**) are from rebar fracture; (**d**–**f**) are from friction among rebar, mortar, and sleeve; and (**g**–**i**) are from concrete crushing.

As discussed in Section 6.2, two damage modes (ductile and brittle damage) correspond to different AE signatures. By extracting the typical signals from different AE events, see Figure 16, three types of AE signals were dominant: (i) For rebar fracture, when the steel bar fracture signal appears, a large number of high-frequency signals with a peak frequency of 260 kHz was recorded, as shown in Figure 17a–c. Usually, the AE signal of metal fracture reflects in a high frequency range from 200 kHz to 400 kHz. Additionally, the signal shows more burst types, see in Figure 17a. In this case, it indicates the fracture of the rebar. (ii) Many low frequency signals were observed during the rebar pull-out. The main frequency component was lower than 100 kHz. It was caused by friction among rebars, mortars, and sleeves. The secondary or third AE phenomenon happened. Usually, the massive low frequency signals with secondary AE are the precursor of the rebar pull-out in the sleeve. For concrete crushing, usually accompanied by the rebar pullout failure, the peak frequency of the signal usually happens at 150 kHz. Other than it, partial AE energy was concentrated in a low frequency range of approximately 50 kHz, which is very similar to the AE signal due to friction. By further analyzing these two AE events, they usually were observed simultaneously. Moreover, concrete crushing and friction were involved during the rebar pull-out process. With the increase in the internal flaw, more AE signals appeared due to concrete crushing and friction. The damage mode tended to be the rebar pullout (brittle mode). It coincided with mechanical behavior and indirectly confirmed the AE findings.

### 6.5. Comprehensive AE and UT Characterization of Damage Status and Processing

**Figure 18:**
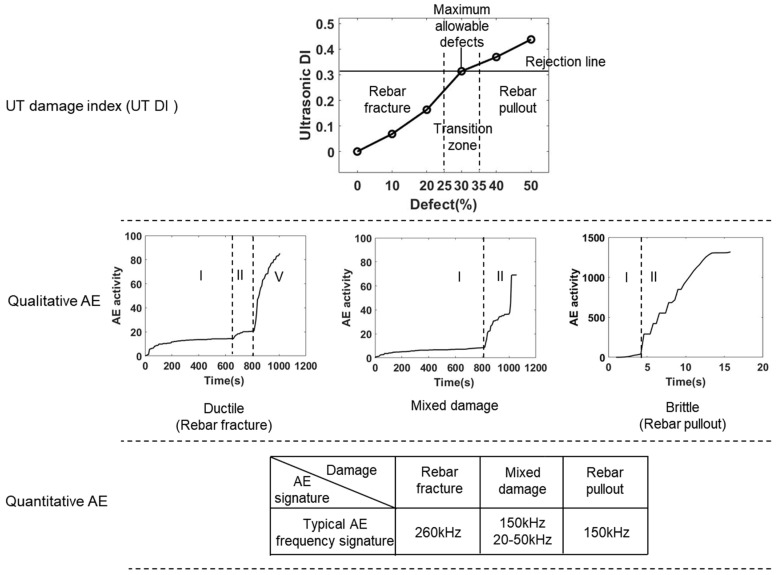
The correlation of the UT damage index, qualitative AE, and quantitative AE.

In this paper, the UT was used to determine the damage status of the grouted sleeve nondestructively; meanwhile, the AE can characterize the damage progress. With the combination of UT and AE, the damage status and its process can be described. For UT, with optimization of the UT configuration and the selection of UT features as mentioned in Section 2, Section 3, Section 4 and Section 5, it is important to define a damage index (DI) correlated to the defect rate. DI is defined based on the UT signal energy ratio from the defect specimen normalized to the UT signal energy ratio obtained from the defect-free sample, and calculated as Equation (4):

(4) $$\mathrm{DI}=\left|\frac{E_{i}-E_{0}}{E_{0}}\right|$$

where $E_{0}$ represents the UT signal energy ratio of the defect-free sample. $E_{i}$ represents the UT signal energy ratio of the defective sample, and i represents the defect rate of the grouting casing. The results of UT DI are shown in Figure 18. With the increase in the volume of the internal flaw, the DI increased. Moreover, it shows a double broken line model. The inflection point was at 30% defect, which corresponds to the DI of 0.31. With consideration of the tensile test, the mixed damage mode (rebar fracture and pullout) happened. The strength of grouted sleeve failed very fast. The damage changed from ductile mode to brittle mode. Additionally, the safety margin was reduced. Therefore, a rejection line was defined as the DI value of 0.31. It means that the UT DI of the sleeve connection above the rejection line, the connection had a great risk in failure due to the effect of internal defects. Thus, for rapid evaluation of UT, the grouted sleeve connector should be disposed as long as the DI is larger than 0.31.

For AE characterization of damage progress, qualitative and quantitative evaluations were conducted, and the results are shown in Figure 18, respectively. In general, if the DI is lower than 0.31, the three stages of damage were observed in AE activities. The damage shows more ductility, and rebar fracture is the main result. Accordingly, the strength of grouted sleeve connector wasn’t impaired by internal flaws. The high frequency AE signals were dominant. On the contrary, once the DI is higher than 0.31, the damage shows more brittleness and the rebar pullout is dominant, which reduces the strength of the grouted sleeve connector and negatively impact the safety. The two stages of AE activities and the frequency of 150 kHz was dominant in AE signals. Approximately DI of 0.31, a transition zone was defined. According to the description in Section 6.2, the specimen with a 30% defect rate is the critical value for rebar fracture failure and rebar pullout failure. Therefore, the 30% defect rate was defined as the maximum allowable defect for bond strength failure of mortar, sleeve, and rebar in grouted sleeves, as shown in Figure 18. By further analyzing the 30% defective sleeve, both ductile damage (plastic deformation of the rebar) and brittle damage can be observed inside the sleeve. To better characterize the damage pattern of the sleeve, the 25–35% defect rate was defined as the transition zone. In this region, both ductile and brittle damage occur inside the sleeve. In this region, the mixed damage modes were observed; and the AE activities show a combined pattern of rebar fracture and pullout. One point needs to be noticed: massive friction signals with low frequency were observed, which is a precursor of failure of the grouted sleeve connector, see the quantitative AE evaluation in Figure 18. 

## 7. Conclusions

In this paper, AE and UT are combined to quantitatively evaluate the internal defects of grouting sleeves and to achieve real-time monitoring of the damage process of grouting sleeves with different degrees of internal defects. The UT was conducted before loading to evaluate the internal flaws. The mechanism of UT detection was comprehensively explained. A linear relationship between the UT signal energy ratio and the grouting defect rate was established through numerical simulations and experimental studies, and the optimal path in UT was determined for detecting defects inside the sleeve. The AE as the processing monitoring technique was used during tensile testing. A qualitative and quantitative AE was created: For rebar fracture damage, AE activity can be divided into three stages; for rebar pullout damage, AE activity can be divided into two stages. The failure of grouting material belongs to brittle failure, and the peak frequency of the signal usually happens at 150 kHz. Rebar fracture is ductile failure, and the peak frequency of the signal happened at 260 kHz. 

This paper studies the horizontal defects of grouting sleeves, which are mainly introduced by the horizontal grouting sleeve connections used in beam-beam members and beam-column member reinforcement joints in PB structures. AE combined with UT, the UT DI was given, and the rejection line was made for in-field rapid evaluation of horizontal grouting sleeve connections. The AE was able to realize the processing evaluation of failure. By using the proposed combined NDE methods, it can provide a concept and general idea for developing a convenient, rapid, and accurate on-site device as a future study. 

The limitation of this study is that the numerical model does not consider the effect of sensors; and also, some experimental uncertainties were ignored in the numerical model, which leads to errors between experimental results and simulation results. In addition, the applicability of the rejection line proposed in this paper was weak due to a lack of repeated testing. More samples with more levels of internal flaw would enhance the foundlings. The only effect of horizontal defects was considered, which limits the universality of the study. Therefore, more sophisticated studies should be conducted in the future.

## Figures and Tables

**Table 1:** Reflection (R) and transmission (T) between all the possible boundaries.

Boundary	Reflection Coefficient (R)	Transmission Coefficient (T)
Sleeve/Flaws	0.85	0.15
Flaws/Mortar	0.49	0.51
Mortar/Rebar	0.63	0.37
Rebar/Mortar	0.63	0.37
Mortar/Sleeve	0.60	0.40

**Table 2:** Material properties in the numerical models.

Materials	Density (kg/m^3^)	Young’s Modulus (GPa)	Poisson’s Ratio
HRB400 (Rebar)	7850	210	0.30
Ductile Iron (Sleeve)	7300	162	0.29
Concrete Mortar (Grout)	2300	20	0.20
Plasticene [45] (Artificial flaw)	1384	0.0031	0.40

**Table 3:** The results of six grouted sleeve connector cyclic loading tests.

Grouted Sleeve Defect Rate	Failure Mode
0%	Rebar fracture
10%	Rebar fracture
20%	Rebar fracture
30%	Rebar pullout
40%	Rebar pullout
50%	Rebar pullout

## Data Availability

The date presented in this study are available on request from the corresponding author.
